# Fast-track care with intraoperative blood salvage in laparoscopic splenectomy

**DOI:** 10.1038/s41598-019-45865-x

**Published:** 2019-07-09

**Authors:** Yan Chen, Jianwei Wang, Qinghuang Ye, Zhijiang Wang, Weihong Weng, Jinhui Zhu

**Affiliations:** 1 Department of General Surgery and Laparoscopic Center, Second Affiliated Hospital, Zhejiang University School of Medicine, 88 Jiefang Road, Hangzhou, 310009 China; 2 Department of General Surgery, Second Affiliated Hospital, Zhejiang University School of Medicine, 88 Jiefang Road, Hangzhou, 310009 China

**Keywords:** Liver cirrhosis, Liver cirrhosis

## Abstract

Fast-track surgery is claimed to reduce medical morbidity, eliminate the hospitalization needs, and shorten the convalescence period. Intraoperative bleeding as the main complication is also the primary cause of conversion from laparoscopic to open splenectomy. Intraoperative blood salvage can reduce transfusion requirements, decrease the conversion rate to open, and promote fast-tracking in laparoscopic splenectomy (LS). From November 2007 through December 2016 we collected medical data of 115 LS patients. There were three groups: 54 patients receiving routine care (we marks them as Group RT), 33 patients with fast-track care (Group FT), and 28 receiving fast-track care receiving intraoperative splenic blood salvage and autotransfusion (Group FT + ISBS). These medical data are comprised of included three phases (pre-, intra-, and postoperative). There were significant differences (*P* < 0.05) between RT, FT, and FT + ISBS groups. The hemoglobin level in Group FT + ISBS was significantly higher than in Group RT and Group FT. Comparing the duration of hospital stay of 3 groups, Group RT stayed for a significantly longer time than Group FT and Group FT + ISBS, Group FT + ISBSmuch shorter than Group FT. Comparing the hospitalization expense, GroupFT + ISBS significantly expended less than Group RT and Group FT. Our study shows that laparoscopic splenectomy with fast-track care is feasible, effective, and safe for patients who require splenectomy. Fast-tracking with intraoperative blood salvage improved the fast-track laparoscopic splenectomy procedure.

## Introduction

Splenectomy has generally been the treatment of choice for splenomegaly and hypersplenism since the 1950s^1^. However, open splenectomy is associated with a high risk of intraoperative hemorrhage and postoperative pain, and it is contraindicated in patients who have poor liver function^2^. Laparoscopic surgery is considered as a minimally invasive technique to reduce the systemic immunologic and inflammatory responses resulting from the surgical trauma of open surgery^3–5^. Laparoscopic splenectomy (LS) was first introduced by Delaitre and Maignien^6^ and has been well studied in patients with splenomegaly due to its promising clinical benefits^7^. In the last 20 years, studies have shown that LS is safe and feasible. There are no significant differences between LS and open splenectomy regarding complications and mortality^8–10^.

Fast-track (FT) surgery was first introduced in 1991 for colorectal surgery^11^. FT surgery is used to enhance the recovery from “single-modality” evidence-based surgical care procedures by applying “a multimodal effort” pattern. FT surgery combines various practices and care regimens in the perioperative period, which include epidural or regional anesthesia, minimally invasive techniques, optimal pain control, preoperative oral nutrition, and postoperative early ambulation^12^. Applying these procedures, fast-tracking can reduce stress responses and organ dysfunction to shorten the recovery time, avoid complications, and decrease cost^13^.

Patel *et al*.^14^ showed that while LS in patients with massive splenomegaly is feasible, it is associated to a longer median operating time, higher conversion rate, higher postoperative morbidity, and longer median postoperative stay. Intraoperative bleeding is the main complication and cause of conversion during LS. Combining LS with intraoperative blood salvage with autotransfusion retains the advantages of autotransfusion and eliminates the risks associated with homologous transfusion.

The evidence for the use of FT with or without intraoperative blood salvage in LS remains incomplete. We performed this study to investigate the application of FT surgery with or without intraoperative blood salvage in LS.

## Materials and Methods

### Patients

This study was conducted in the General Surgery Department, Zhejiang University School of Medicine Second Affiliated Hospital, China. From November 2007 through December 2016, a retrospective review of medical charts was performed of patients who underwent LS by the same surgeons’ treatment team. Totally 115 patients involved in this study, 54 patients received routine care (group RT), 33 received FT care (group FT), and the rest 28 received both FT care and intraoperative splenic blood salvage (group FT + ISBS). The data included 3 perioperative (pre-, intra-, and postoperative) phases (Table 1). The data were collected from both electronic and hard copy databases. Before the study took place, the ethics committee (Department of Ethics committee, Zhejiang University School of Medicine Second Affiliated Hospital) reviewed and approved the study protocol, and submitted these data of the study to the ethics committee. At the same time, we got approved and signed the agreements from all the subjects (if the subject is under 18, from his/her parent and/or the legal guardian). All methods were carried out in accordance with relevant guidelines and regulations. Informed consent has been obtained from all subjects, or parents/legal guardians of subjects under 18 years old. All the subjects (or parents/legal guardians of subjects under 18 years old) understood the consent and inclusion of informed consent, and agreed. The selection criteria of these patients is based on the injurious mechanism as hypersplenism with mild to severe splenomegaly; liver function with Child-Pugh class A or B, one or more episodes of bleeding after a course of endoscopic sclerotherapy or banding, medical treatment failed,variceal bleeding proved and assessed by endoscopy and variceal bleeding due to cirrhosis caused by any etiology.

**Table 1:** Patient characteristics by surgery groups.

Preoperative phase Patients age, gender, Child-Pugh class (A:B), spleen longitudinal diameter (cm), perioperative blood test included haemoglobin (Hb), AST, ALT.
Intraoperative phase operation time (minites), volume of intraoperative blood loss and blood autotransfusion, the numbers of conversion to open
Postoperative phase the numbers of postoperative fever (>38.5 °C), the others complications including pulmonary infection, tender pancreatic leakage, subphrenic abscess, injure of colon, postoperative blood test included haemoglobin (Hb), Tbil, Ibil, AST, ALT, Hb increase, hospital stay, hospitalization expenses.

### LS

This study team has 20 years experience in LS performing, the surgery procedures: After general anesthesia administration and endotracheal intubation, patients were placed in the right lateral decubitus and 60° semilateral position. Four trocars, one with a diameter of 12 mm, two 10 mm, and one 5 mm were placed with CO_2_ pneumoperitoneum created to 12 to 14 mmHg^15^. The first trocars were positioned with a 10-mm trocar in the umbilical position for the 30° camera, a 5-mm trocar in the subxiphoid epigastrium for the retractor, and a 10-mm trocar in the left subcostal arch for the LigaSure vessel-sealing equipment (Covidien/Medtronic, Boulder, CO, USA) or shears. The 12-mm trocar was positioned in the left midaxillary line halfway between the left costal margin and the iliac crest or below the border of the spleen for the use of the endoscopic linear vascular stapling device or other supplementary device.

Using LigaSure instruments, mobilization of the spleen was performed inferiorly. After that, the splenocolic attachments and the gastrocolic ligament were divided and opened to expose the lesser sac. The gastrosplenic ligament (including the short gastric vein) and the splenorenal ligament were dissected and coagulated carefully and then cut. The splenic hilum was raised, and the splenectomy was completed while cutting the splenic artery and vein using an endoscopic linear vascular stapler (Echelon 60, 2.5 mm; Ethicon Endo-Surgery, Inc., Cincinnati, Ohio, USA). The spleen was then freed and placed into a hard plastic bag to prevent spillage of blood or splenic pulp into the abdominal cavity.

### Fast-track surgery

FT surgery includes various techniques that are also used in the care of patients undergoing LS (Table 2)^16^. In this study, these specific techniques were applied to FT and FT + ISBS groups. The control group underwent conventional laparoscopy.

**Table 2:** Perioperative care in FT surgery.

Preoperative Phase Preoperative risk assessment for the special surgery Information to all the relative nurses, doctors and patients Fluid optimization
Alcohol/smoking abstinence No bowel preparation Modern fasting guidelines:oral glucose fluid 2 h before surgery
Intraoperative Phase Fluid optimisation (avoid hypovolaemia and crystalloid excess) :2000ml-2500ml/day in the day of surgery Regional anaesthesia:transabdominal pain block before the end of general anaesthesia Type of incision:horizontal Short-acting opioids
Postoperative Phase Multimodal analgesia:intravenous, by mouth. Anti-emetic and anti-ileus prophylaxis Revise use of drains, tubes, catheters, monitoring, etc. Thromboembolic prophylaxis Early oral nutrition and ambulation:drink water 2 h after surgery;fluid and ambulation in the day one after surgery Daily care maps, well-defined discharge criteria Rehabilitation plan

### Intraoperative spleen blood salvage

**Figure 1:**
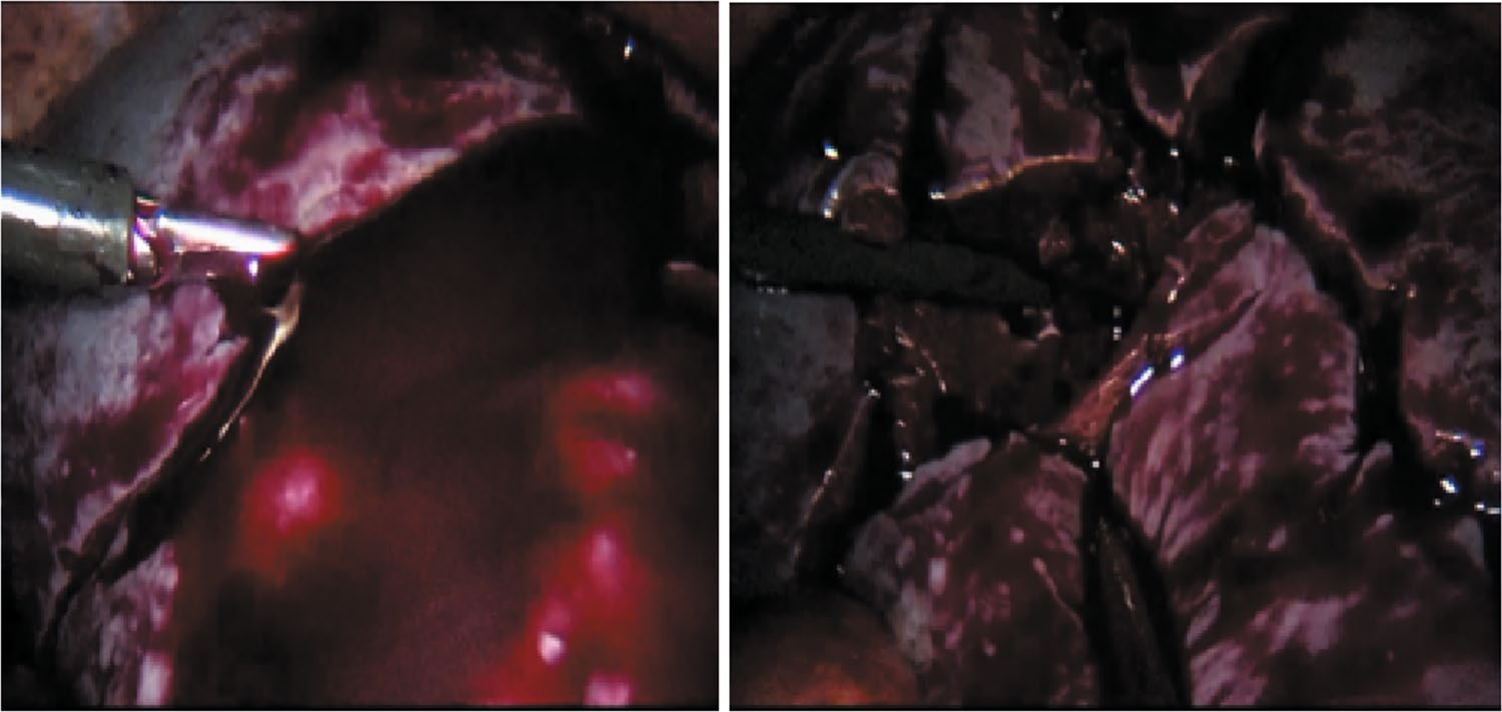
Blood collected from the freed spleen in a hard bag were transferred to an autotransfusion device via a vacuum extractor.

Patients in the FT + ISBS group underwent intraoperative cell salvage. Blood collected from the operative field or the freed spleen in a hard bag were transferred to an autotransfusion device (Autologous blood recovery system, Jingjing Medical Equipment Co. Ltd., Beijing, China) using a double-tube suction device (Fig. 1). The RBCs were washed and filtered by the autologous equipment, while the WBCs, platelets, plasma, and heparin were removed. The salvaged RBCs were transfused back into patients over a 6-h period.

### Statistical analysis

Other analysis variables included the patient characteristics, intraoperative parameters, and postoperative course. Numerical data were expressed as mean ± standard deviation (SD). Group means were compared using Student’s t-test (for data with a normal distribution) or the Mann–Whitney U-test (for data with nonnormal distribution), and the Chi-square test was used to compare percentages. A *P* value < 0.05 was considered statistically significant. IBM SPSS Statistics for Windows, Version 19.0 (IBM Corp., Armonk, NY, USA) was used for statistical analysis.

## Results

### Preoperative patient characteristics

As shown in Table 3, there were 54 patients in the RT group, 33 patients in the FT group, and 28 patients in the FT + ISBS group. Comparing these three groups, there were demographical similarities, age, gender, Child-Pugh class, and spleen longitudinal diameter, as well as, hemoglobin (HB), total bilirubin (TBil), indirect bilirubin (IBil), aspartate transaminase (AST), and alanine transaminase (ALT) levels.

**Table 3:** Patient characteristics by surgery groups.

Treatment group	RT	FT	FT + ISBS	*P* ^*α*^	*P* ^*β*^	*P* ^*γ*^
Number of patients, n	54	33	28	/	/	/
Age, yr (mean ± SD)	43.5 ± 6.7	47.1 ± 7.3	45.4 ± 5.5	NS	NS	NS
Gender ratio,(F:M)	34:20	23:10	19:9	NS	NS	NS
Child-Pugh class (A:B)	19:35	8:24	6:22	NS	NS	NS
Spleen longitudinal diameter, cm	20.4 ± 3.7	21.8 ± 4.5	22.7 ± 5.2	NS	NS	NS
Hb, g/L	9.6 ± 0.9	9.3 ± 1.1	8.9 ± 1.4	NS	NS	NS
TBil, μmol/L	36.5 ± 7.6	40.6 ± 7.3	37.9 ± 8.7	NS	NS	NS
IBil, μmol/L	27.3 ± 5.7	30.1 ± 7.7	28.5 ± 7.9	NS	NS	NS
AST, U/L	235.2 ± 93.6	259.7 ± 80.5	190.1 ± 100.1	NS	NS	NS
ALT, U/L	472.1 ± 174.5	500.1 ± 220.5	450.6 ± 191.2	NS	NS	NS

Haemoglobin = Hb; F = female; M = male;TBil = Total bilirubin; IBil = Indirect bilirubin; AST = glutamic oxalacetic transaminase; ALT = glutamic-pyruvic transaminase; NS = not significant.

*P*^*α*^ FT group comparring to TR group, <0.05 has statistical significance*;

*P*^*β*^FT + ISBS group comparring to RT group, <0.05 has statistical significance†;

*P*^*γ*^FT + ISBS group comparring to FT group, <0.05 has statistical significance§.

### Operative data

Comparing these three groups, there were no significant differences in operative time, blood loss, volume of blood autotransfused, and intraoperative complications (Table 4).

**Table 4:** Operative and complication data.

Treatment group	RT	FT	FT + ISBS	*P* ^*α*^	*P* ^*β*^	*P* ^*γ*^
Operative time, h	2.8 ± 0.7	3.0 ± 1.1	2.9 ± 0.9	NS	NS	NS
Blood loss, ml	95.0 ± 40.5	100.5 ± 35.5	80.5 ± 32.5	NS	NS	NS
Volume of blood autotransfused (ml)	0	0	500.2 ± 160.5	/	/	/
Conversion to open, n (n%)	2 (3.7%)	0	1 (3.6%)	NS	NS	NS
**Complications**						
Postoperative fever (>38.5 °C), n (n%)	9 (16.7%)	4 (12.1%)	5 (17.9%)	NS	NS	NS
Others, n (n%)	4 (7.4%)	2 (6.0%)	1 (3.61%)	NS	NS	NS

Others including pulmonary infection, tender pancreatic leakage, subphrenic abscess, injure of colon.

*P*^*α*^ FT group comparring to TR group, < 0.05 has statistical significance*;

*P*^*β*^FT + ISBS group comparring to RT group, < 0.05 has statistical significance†;

*P*^*γ*^FT + ISBS group comparring to FT group, < 0.05 has statistical significance§.

#### Postoperative blood tests and complications

The Hb level in the FT + ISBS group was (11.2 ± 1.1) g/Lafter surgery, which was significantly higher than (9.3 ± 0.7) g/Lin the RT group and (9.4 ± 0.9) g/L in the FT group, showing a significant difference (*P* < 0.05). Furthermore, the increase in Hb in the FT + ISBS group was (2.3 ± 1.0) g/L at postoperative day one, which was significantly higher than (0.5 ± 0.2) g/Lin the RT and (0.6 ± 0.4) g/Lin the FT groups. At postoperative day three, the Hb increase in the FT + ISBS group was (2.5 ± 0.8) g/L, which was significantly higher than (0.6 ± 0.3) g/L in the RT and (0.5 ± 0.2) g/L in the FT groups. There were significant differences (*P* < 0.05) between the groups without splenic blood autotransfusion and the FT + ISBS group. Meanwhile, the other blood test data (TBil, IBil, AST, and ALT) were not statistically different between the groups. The postoperative blood test data are shown in Table 5.

**Table 5:** Postoperative data of surgery groups.

Treatment group	RT	FT	FT + ISBS	*P* ^*α*^	*P* ^*β*^	*P* ^*γ*^
Hb, g/L	9.3 ± 0.7	9.4 ± 0.9	11.2 ± 1.1	NS	<0.05†	<0.05§
TBil, μmol/L	37.5 ± 5.6	39.2 ± 7.1	38.2 ± 6.5	NS	NS	NS
IBil, μmol/L	27.8 ± 5.1	29.8 ± 6.7	29.6 ± 7.7	NS	NS	NS
AST, U/L	255.2 ± 99.2	249.2 ± 86.1	220.1 ± 80.8	NS	NS	NS
ALT, U/L	452.1 ± 175.3	488.7 ± 251.4	430.6 ± 186.5	NS	NS	NS
Hb increase (1d), g/L	0.5 ± 0.2	0.6 ± 0.4	2.3 ± 1.0	NS	<0.05†	<0.05§
Hb increase (3d)	0.6 ± 0.3	0.5 ± 0.2	2.5 ± 0.8	NS	<0.05†	<0.05§
Hospital stay, d	8.4 ± 2.2	6.7 ± 2.8	5.4 ± 1.3	<0.05*	<0.05†	<0.05§
Hospitalization expenses, RMB (thousand)	66.5 ± 21.5	53.5 ± 16.8	41.2 ± 13.6	<0.05*	<0.05†	<0.05§

Haemoglobin = Hb; Hb increase = Hb (postoperaion) - Hb (preoperation); F = female; M = male; TBil = Total bilirubin; IBil = Indirect bilirubin; AST = glutamic oxalacetic transaminase; ALT = glutamic-pyruvic transaminase; NS = not significant.

*P*^*α*^ FT group comparring to RT group, <0.05 has statistical significance*;

*P*^*β*^FT + ISBS group comparring to RT group, <0.05 has statistical significance†;

*P*^*γ*^FT + ISBS group comparring to FT group, <0.05 has statistical significance§.

Fever was the most common complication after LS. Nine patients of RT group had fever (temperature > 38.5 °C), while four in the FT group, and five in the FT + ISBS group had fever after operation. There are other complications including pulmonary infection, pancreatic leakage, subphrenic abscess, and injuryto the colon. Four patients in the RT group, two patients in the FT group, and one patient in the FT + ISBS group experienced one of these complications. All complications were managed by conservative treatment, no patients died in the perioperative period. There were no significant differences in postoperative data among the three groups.

#### Hospitalization data

Our discharge criteria included the following parameters:postoperative pain adequately controlled with oral analgesics, no need for intravenous nutrition or fluids, mobilization (out of bed ≥ 6 h daily), recovery of bowel function (stool or repeated flatus), and no complications requiring hospital treatment. The hospital stay of RT group was (8.4 ± 2.2) days after surgery, which was significantly longer than (6.7 ± 2.8) days of FT group and (5.4 ± 1.3) days of FT + ISBS group, showing a significant difference (*P* < 0.05). The median length of the hospital stay of patients in FT + ISBS group was (5.4 ± 1.3) days after surgery, which was significantly shorter than (6.7 ± 2.8) days in FT group, a significant difference (*P* < 0.05). The hospitalization expense of FT + ISBS group was (41.2 ± 13.6) thousand RMB, which was significantly less than that of RT (66.5 ± 21.5) and FTgroups (53.5 ± 16.8).

## Discussion

LS is considered as the golden standard for splenectomy for a normal to moderately large spleen. In the early development of this procedure, a study by Patel *et al*.^14^ showed that LS in patients with massive splenomegaly is feasible but associated with a longer median operative time, higher conversion rate, higher postoperative morbidity, and higher median postoperative stay^7^. With the evolution of laparoscopic skill and technology, LS in patients with massive splenomegaly is also safe, as has been verified in recent studies^8,17^. The study team has been performing LS for more than 20 years, and has published two papers demonstrating the feasibility and safety of LS with intraoperative blood autotransfusion. Our study data shows no significant differences of the following items, spleen longitudinal diameter, operation time, blood loss, conversion to open, postoperative complications. This fully proves that our LS performing skills are mature and hence the stable outcomes. Studies^18^ described the learning curve should be of LS of minimum 20 cases which is far less than the amount we performed.

There are many studies on FT surgery, which is widely used in conjunction with laparoscopic surgery, with reports indicating significantly shorter postoperative hospital stays and less expensive hospitalizations^19–21^. LS is a minimally invasive technique, reducing the systemic immunologic and inflammatory responses, and an important concept in FT surgery. However, the safety of fast-tracking used in LS is unknown. Our surgery department has published two studies demonstrating the feasibility and safety of LS with intraoperative blood autotransfusion^17,22^.

This study differs from our earlier studies and those by others in the application of FT LS with or without intraoperative blood autotransfusion. Firstly, there are few studies of standard procedures in FT LS. Secondly, we intended to explore the value in applying intraoperative blood autotransfusion which has been used in FT surgery procedures for LS. Moreover, we conjectured that using intraoperative blood autotransfusion could increase the confidence of the surgeon. Finally, our study shows that fast-tracking plus intraoperative blood autotransfusion is a feasible, safe, and useful procedure for LS.

In this study, the hospital stay for patients in the RT group was (8.4 ± 2.2) after surgery, and the expense was (66.5 ± 21.5) thousand yuan. The hospital stays and expenses of the FT and FT + ISBS groups (the FT group: 6.7 ± 2.8 days and 53.5 ± 16.8 thousand yuan, the FT + ISBS group: 5.4 ± 1.3 days and 41.2 ± 13.6 thousand yuan) were significantly less than those of the RT group. Fast-tracking was very important in reducing the stress responses and organ dysfunction, which led to a decreased recovery time, fewer complications, and less cost. Information about FT LS was provided not just to patients but to all concerned. The patients who underwent FT surgery were well prepared and acknowledged with all procedures^23^. That makes them relaxd and reduce the stress that can increase the systemic immunologic and inflammatory responses. Nurses who were well informed and experienced could also relieve patient anxiety and fear as well as obtain trust and cooperation from patients. A study by Gustafsson *et al*. showed that the hospital stays of patients with high FT protocol adherence is significantly shorter than those with low adherence^24^.

Other preoperative elements of FT surgery include fluid optimization, alcohol and smoking abstinence, oral glucose fluid intake 2 hour before surgery, and no bowel preparation, all of which could reduce postoperative insulin resistance^25^, improve patient well-being (less thirst, hunger, and anxiety) preoperatively^26^, and lower the risk of postoperative fluid overloading and conditions delaying discharge^27^.

In the intraoperative period, using less intraoperative grainage^28^, epidural analgesia by anesthetists can effectively relieve the patients’ pain. There are other ways to control postoperative pains including self-control venous analgesic pumps, intravenous analgesics, and oral analgesics, depending on the patient’s needs. Good pain control can reduce the length of the hospital stay after surgery and therefore decrease hospitalization expenses.

With good pain control, patients in the FT and FT + ISBS groups were encouraged to move on the day of surgery and get up from bed on postoperative day 1. These procedures can reduce the risk of complications such as deep venous thrombosis, because the platelet level in patients who have undergone splenectomy is much higher than the preoperative level, even more than 1000e9/L in some hypersplenism patients with splenomegaly, increasing the risk of thrombosis.

FT applied to LS promotes patient rehabilitation. In our study, we found that the hemoglobin level in the FT + ISBS group was significantly higher than in the RT and FT groups. This result held true for the postoperative hemoglobin determinations on days 1 and 3 after surgery. In other words, there were significant differences between the patients who did not receive autotransfused blood intraoperatively and the FT + ISBS group of patients who did. Autotransfusion in patients in the FT + ISBS group played an important role in increasing the hemoglobin level, which is important for patients of variceal bleeding due to cirrhosis, and thus reducing symptoms such as the dizziness and fatigue, all of which can lower the patients’ willingness to be discharged.

Bleeding is the main intraoperative complication of LS and the main reason to convert to open surgery, especially for the splenomegaly patients, although LS is known to be safe. Conversion to open splenectomy should be considered when intraoperative bleeding cannot be managed promptly^28^. The conversion to open splenectomy in this situation is easier with intraoperative blood autotransfusion. Furthermore, there are additional potential postoperative complications that come into play if the surgery is converted to open splenectomy^28^. Examples of these complications are delays in pain control, mobilization, and resumption of oral nutrition, which are not achieved easily.

The risk of intraoperative bleeding in splenomegaly with or without portal hypertension from liver cirrhosis is high^29^. This comes from conditions such as low platelet level, impaired coagulation factors, splenomegaly, and the development of collateral hepatic vessels. In our study, blood from the operative field and resected spleen were recovered by an autologous blood recovery system. In LS, intraoperative splenic blood autotransfusion provides the advantages of autotransfusion and eliminates the risks associated with homologous transfusion. Intraoperative blood autotransfusion is relatively easy on a technical level and has the advantage of significantly increased postoperative hemoglobin levels, as described earlier.

Therefore, we believe that FTLS with intraoperative autotransfusion is worthy of consideration and clinical application.

## Conclusion

FT LS is feasible, providing effective and safe surgical care for patients who require splenectomy. FT LS with intraoperative blood autotransfusion has the advantage of further LS improvement, especially for patients with potentially massive intraoperative bleeding or rare blood types (such as Rh negative), or in patients with religious concerns related to blood transfusion. Our study showed that additional improvements in FT surgery can be developed and applied optimizing both the procedure and patient outcomes.
